# Molecular Characterization of *Staphylococcus aureus* Plasmids Associated With Strains Isolated From Various Retail Meats

**DOI:** 10.3389/fmicb.2020.00223

**Published:** 2020-02-19

**Authors:** Leena Neyaz, Nisha Rajagopal, Harrington Wells, Mohamed K. Fakhr

**Affiliations:** Department of Biological Science, The University of Tulsa, Tulsa, OK, United States

**Keywords:** *Staphylococcus*, *Staphylococcus aureus*, plasmid, retail meats, replicon, PFGE, megaplasmids, antimicrobial resistance

## Abstract

*Staphylococcus aureus* is considered one of the most important foodborne bacterial pathogens causing food poisoning and related illnesses. *S. aureus* strains harbor plasmids encoding genes for virulence and antimicrobial resistance, but few studies have investigated *S. aureus* plasmids, especially megaplasmids, in isolates from retail meats. Furthermore, knowledge about the distribution of genes encoding replication (*rep*) initiation proteins in food isolates is lacking. In this study, the prevalence of plasmids in *S. aureus* strains isolated from retail meats purchased in Oklahoma was investigated; furthermore, we evaluated associations between *rep* families, selected virulence and antimicrobial resistance genes, and food source origin. Two hundred and twenty-two *S. aureus* isolates from chicken (*n* = 55), beef liver (*n* = 43), pork (*n* = 42), chicken liver (*n* = 29), beef (*n* = 24), turkey (*n* = 22), and chicken gizzards (*n* = 7) were subjected to plasmid screening with alkaline lysis and PFGE to detect small-to-medium sized and large plasmids, respectively. The *S. aureus* isolates contained variable sizes of plasmids, and PFGE was superior to alkaline lysis in detecting large megaplasmids. A total of 26 *rep* families were identified by PCR, and the most dominant *rep* families were *rep*_10_ and *rep*_7_ in 164 isolates (89%), *rep*_21_ in 124 isolates (56%), and *rep*_12_ in 99 isolates (45%). Relationships between selected *rep* genes, antimicrobial resistance and virulence genes, and meat sources were detected. In conclusion, *S. aureus* strains isolated from retail meats harbor plasmids with various sizes and there is an association between *rep* genes on these plasmids and the meat source or the antimicrobial resistance of the strains harboring them.

## Introduction

*Staphylococcus aureus* was discovered by the surgeon Sir Alexander Ogston in sepsis and abscess (Ogston, 1882), and has continued to be one of the most prominent and well-studied human pathogens in hospital and community infections. *S. aureus* occurs in the microflora of humans and animals, both on the skin and in the respiratory tract; however, it is also an opportunistic pathogen causing diseases that vary widely in severity (DeLeo and Chambers, 2009).

In the United States, *S. aureus* is one of the top foodborne pathogens and causes an estimated 241,000 illnesses per year (Scallan et al., 2011). Many food products become contaminated with *S. aureus* due to its ability to tolerate and grow under different stressful environments (Kadariya et al., 2014), and this can result in staphylococcal food poisoning due to the production of *S. aureus* enterotoxins (Argudín et al., 2010). On the other hand, multiple studies in the US have revealed a high prevalence of multidrug-resistant *S. aureus* strains (MDR) in retail meats (Waters et al., 2011; O’Brien et al., 2012; Jackson et al., 2013) indicating the potential threat for acquisition of virulent strains by meat industry workers. Furthermore, contaminated meat consumers might be at risk for MDR *S. aureus* colonization, and in rare cases may develop severe infections (Kluytmans et al., 1995) or act as healthy carriers for *S. aureus* transmission (Fritz et al., 2009). The meat production process can also contribute to the contamination of retail meats via workers, food animals, meat processing surfaces and equipment (Kadariya et al., 2014).

In the food chain, horizontal gene transfer (HGT) of mobile genetic elements (MGEs) is the most significant mechanism for exchange of antimicrobial resistance and virulence determinants in bacterial populations (Aarestrup et al., 2008). In *S. aureus*, MGEs can be categorized into plasmids, transposons, insertion sequences, bacteriophages, pathogenicity islands, and chromosomal cassettes (Malachowa and DeLeo, 2010). *S. aureus* plasmids can confer resistance to antimicrobials, biocides, and heavy metals (Jensen and Lyon, 2009) and may encode host survival elements, virulence factors, and toxins (Malachowa and DeLeo, 2010). The acquisition of these different genetic elements in a single large plasmid can enhance adaptation and dissemination of *S. aureus* in different environments due to co-selective advantage (Wall et al., 2016). A recent study investigating ST239 MRSA strains evolution over the last 32 years revealed that plasmids coevolved with strains and enhanced resistance to multiple antibiotics (Baines et al., 2019).

The classification of plasmids by replicon typing has been a useful tool to investigate the dynamics of these molecules within bacterial populations in different ecological niches (Orlek et al., 2017). This system originally classified plasmids *in vitro* according to incompatibility (Inc.) groups, which is defined as the inability of plasmids with the same replication machinery to be hosted by the same bacterial cell (Novick, 1987). Inc. groups have been identified in *Enterobacteriaceae* (*n* = 27 Inc. groups), *Pseudomonas* (*n* = 14), and *Staphylococcus* (*n* = 18) (Shintani et al., 2015). PCR based replicon typing (PBRT) methods that target different replicon sequences have replaced the classic Inc. scheme. More recently, a PBRT scheme was developed for enterococci and staphylococci, which included 26 *rep* families and 10 unique families (Jensen et al., 2010; Lozano et al., 2012).

Despite years of study, our current understanding of plasmids and how they are distributed within *S. aureus* populations remains lacking. Most studies that have characterized *S. aureus* plasmids have been biased toward clinical strains, especially MRSA (Caddick et al., 2005; Kadlec and Schwarz, 2010; Shahkarami et al., 2014); whereas, those focusing on food or retail meats are extremely limited with respect to the role of extrachromosomal DNA. Furthermore, the ambiguity surrounding plasmid distribution in different species is likely due to inconsistent replicon typing schemes. Shintani et al. (2015) reported that only 8.4% of publicly available plasmids could be classified by the 26 *rep* families, and only few plasmids were classified by both *rep* family and Inc. group. Additionally, studies that used *rep* families for investigating *S. aureus* plasmids were biased toward European geographical origin and did not include diverse sources. For example, Lozano et al. (2012) investigated 92 *S. aureus* isolates originating from Spain and Denmark; only five isolates were from food sources (pigs). In another study, the sequences of 243 *S. aureus* plasmids available from the public domain were analyzed, but the source of the strains harboring the plasmids was largely unknown (McCarthy and Lindsay, 2012).

The aims of this study were several-fold. One objective was to determine the prevalence of plasmids in 222 *S. aureus* strains isolated from various Oklahoma retail meats. The aim was to understand how *S. aureus* plasmids are distributed in various ecological niches that are different from human and animal isolates. Secondly, the 222 strains were screened for various *rep* families to determine plasmid diversity. Finally, we sought to investigate possible links between *rep* families, retail meat origin, and antimicrobial resistance.

## Materials and Methods

### Strains Used for Plasmid Isolation

A total of 222 *S. aureus* strains were used in this study and were previously isolated from the following retail meats: chicken (*n* = 55), beef liver (*n* = 43), pork (*n* = 42), chicken liver (*n* = 29), beef (*n* = 24), turkey (*n* = 22), and chicken gizzards (*n* = 7). These strains were previously isolated and screened for antimicrobial susceptibility to 16 different antibiotics (Abdalrahman et al., 2015a, b; Abdalrahman and Fakhr, 2015). Resistance of these 222 *S. aureus* strains to the following twelve antimicrobials (azithromycin, ciprofloxacin, gentamicin, oxacillin, tetracycline, vancomycin, trimethoprim/sulfamethazole, clindamycin, penicillin, erythromycin, rifampin, and chloramphenicol) were reassessed following the most recent CLSI published breakpoints to determine any possible association with rep types (CLSI, 2019).

### Isolation of Small Plasmids by Alkaline Lysis

Plasmids were isolated using a modified midi-preparation method as described previously for small plasmids (<60 kb) (Sambrook and Russell, 2001). Briefly, cells were grown in 15–20 ml of Luria Broth (LB) (Becton Dickinson, Sparks, MD, United States) with agitation at 200 rpm at 37°C for 12–16 h. Cells were harvested by centrifugation at 5,000 rpm for 6 min, and pellets were re-suspended in 500 μl Tris-EDTA buffer (TE) (10 mM Tris, 1 mM EDTA, pH 8) (Amresco, Solon, OH, United States) and 3 μl of lysostaphin stock solution (1 mg/ml lysostaphin in 20 mM sodium acetate, pH 4.5) (Sigma-Aldrich, St. Louis, MO, United States). After a 30-min incubation at 37°C in the water bath, 6 ml of alkaline lysis solution of pH 12.4 (TE buffer containing 0.1 N NaOH and 0.5% SDS) was added and mixed by inversion until the cell suspension was clear. A 3 ml solution of 3.0 M sodium Acetate, pH 5.2 (Sigma-Aldrich) was added, and the suspension was centrifuged at 6,000 rpm for 10 min at room temperature. The supernatant was then transferred to a fresh 50 ml tube, mixed with 9 ml of isopropanol and centrifuged for 30 min at 10,000 rpm. After the supernatant was discarded, the plasmid DNA pellet was rinsed with 70% ethanol (5 ml), resuspended in 250 μl of TE buffer and then stored at −20°C. Plasmids were electrophoretically separated in 0.8% agarose gels (VWR, Radnor, PA, United States) at 120 V for 2.75 h; *E. coli* strains NCTC 50192 and 50193 and a 100 bp DNA ladder (Bioneer corporation, Alameda, CA, United States) were used as references and size standards, respectively. Gels were stained with ethidium bromide, and images were captured using a Bio-Rad Gel DocTM XR UV gel documentation system (Bio-Rad, Hercules, CA, United States).

### Detection of Large Plasmids by Pulsed Field Gel Electrophoresis

Large plasmids (>60 kb) were screened by PFGE using protocols supplied by the CDC Pulse Net (McDougal et al., 2003; Marasini and Fakhr, 2014). Strains were grown in Typtic Soy Agar (TSA) (Himedia, Mumbai, India) at 37°C for 16–18 h, harvested by centrifugation, and resuspended in cell suspension buffer (CSB) (100 mM Tris, 100 mM EDTA, PH 8.0) to an OD of 0.9–1.1 at 610 nm. The adjusted cell suspension (200 μl) was centrifuged at 13,000 rpm for 3–4 min, the supernatant was decanted, and the pellet was resuspended in TE buffer (300 μl). The adjusted cell suspension incubated at 37°C for 10 min and then supplemented with 4 μl lysostaphin stock solution and 300 μl of 1.8% SeaKem Gold agarose (Lonza, Allendale, NJ, United States) in TE buffer (equilibrated to 55°C). This mixture was dispensed into the wells of plug molds and allowed to solidify at room temperature for 10–15 min. Plugs were then removed and transferred to a tube containing 3 ml of EC lysis buffer (6 mM Tris HCl, 1 M NaCl, 100 mM EDTA, 0.5% Brij 58, 0.2% sodium deoxycholate, 0.5% sodium lauroylsarcosine). After a 4-h incubation at 37°C, the EC lysis buffer was decanted and TE buffer (4 ml) was added; tubes were then agitated for 30 min at room temperature. The buffer was removed, and the washing was repeated three more times; after the final wash, 4 ml of TE buffer was added and samples were stored at 4°C. A small section was excised from the plugs and linearized with S1 nuclease as described previously (Marasini and Fakhr, 2014). The plugs were inserted into 1% agarose wells in TBE buffer and electrophoresed with *Xba*I Salmonella serovar Braenderup H9812 for 14 h in 0.5 X TBE as described (Marasini and Fakhr, 2014). Gels were stained and DNA bands were visualized as described above.

### PCR Analysis of *rep* Genes

Total DNA was extracted by single cell lysing buffer (SCLB) as described previously (Marmur, 1961; Noormohamed and Fakhr, 2012). One colony from each TSA plate was mixed with 40 μl SCLB containing 1 ml of TE and 10 μl of 5 mg/ml proteinase K (Amresco, Solon, OH, United States). This mixture was incubated for 10 min at the following temperatures; 80, 55, and 95°C. Mixtures were then diluted (1:2) in double distilled water (80 μl) and centrifuged for 1 min at 4500 rpm. DNA samples were then screened for *rep* genes using seven multiplex PCRs for 26 different primers (Integrated DNA Technologies, Coralville, IA, United States) (Supplementary Table S1). These primers targeted *rep* genes of defined plasmid groups previously detected in Gram positive taxa (Jensen et al., 2010; Lozano et al., 2012). PCR reactions consisted of the following: Master Mix, 10 μl; sterile distilled water, 4 μl; primers, 1 μl each, and DNA template, 2 μl. Multiplex PCR was conducted as described (Abdalrahman and Fakhr, 2015), and stored at −20°C after PCR. Multiplex PCR products (10 μl) were loaded into 2% agarose gels containing 1X TAE buffer and separated by electrophoresis (140 V, 80 min); gels were stained and DNA bands were visualized as described above.

### Statistical Analysis

A Chi-Square test of independence was performed to evaluate associations between plasmid *rep* families and meat sources (Sokal and Rohlf, 1995). This test posits that the occurrence of a rep-type in a meat-type is simply the relative-frequency of a particular rep-type × the relative-frequency of *rep*s in particular meat type (multiplied by the sample size). The statistic calculation is that of any chi-square (*X*^2^ = ∑(*o_i_*−*e_i_*)^2^/*e_i_*) where *o_i_* = observed cell value and *e*_*i*_ = expected cell value). This tests limits the number of cells that can have very small expected values, so we eliminated rep-types that were very rare (1, 3, 9, 10b), and the meat type with very few samples (chicken gizzards).

## Results

### Prevalence of Plasmids in *S. aureus* Strains

**FIGURE 1:**
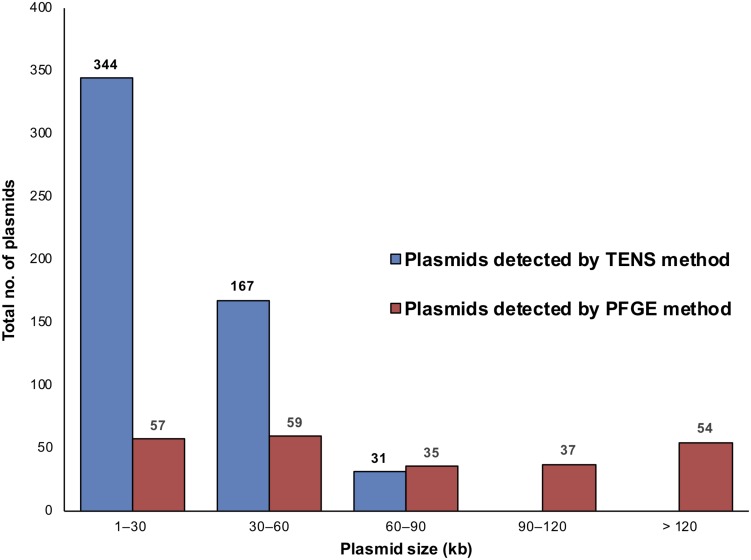
The distribution of plasmids in the 222 *S. aureus* isolates according to plasmid size and method of detection, TENS and PFGE. Each method type is denoted by a different color, as shown in the color code at the right. The horizontal axis distributes plasmids according to the size windows shown. The vertical axis denotes the number of plasmids in each size window. Here, more plasmids were seen than the number of isolates as some isolates contained multiple plasmids.

The plasmid content of 222 *S. aureus* isolates (55 chicken, 43 beef liver, 42 pork, 29 chicken liver, 24 beef, 22 turkeys, and 7 chicken gizzard) was analyzed using both alkaline lysis and PFGE (Table 1). PFGE was helpful in detecting large plasmids (Supplementary Figure S1), which were generally undetectable using the alkaline lysis method (Figure 1). For instance, among the 222 *S. aureus* isolates, alkaline lysis method (TENS) detected 542 plasmids smaller than 90 kb while PFGE identified only 151 plasmids within that size range. On the other hand, PFGE identified 91 plasmids larger than 90 kb. None of these plasmids have been detected by TENS method (Figure 1).

**TABLE 1:** The prevalence of plasmids in *S. aureus* isolated from various retail meats by alkaline lysis (TENS) and PFGE.

**Source**	**No. of isolates**	**No. of isolates with plasmids**			
		**Detected by TENS**		**Detected by PFGE**	
		**2–60 kb**	**>60 kb**	**2–60 kb**	**>60 kb**
Chicken	55	55(100%)	4(7.3%)	30(54.0%)	27(49.1%)
Beef liver	43	43(100%)	4(9.3%)	11(25.6%)	19(44.2%)
Pork	42	42(100%)	7(16.7%)	11(26.2%)	19(45.2%)
Chicken liver	29	29(100%)	3(10.3%)	13(44.8%)	15(51.7%)
Beef	24	24(100%)	2(8.3%)	6(25.0%)	8(33.3%)
Turkey	22	22(100%)	10(45.4%)	15(68.2%)	12(54.5%)
Chicken gizzard	7	7(100%)	1(14.3%)	2(28.6%)	1(14.3%)
Total	222	222(100%)	31(14.0%)	88(39.6%)	101(45.5%)

**FIGURE 2:**
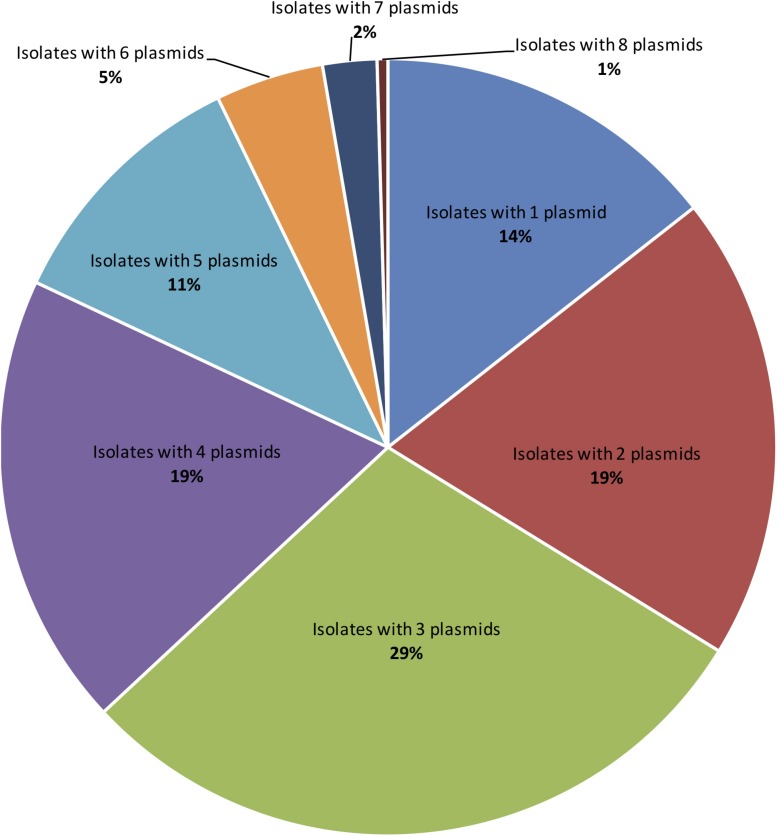
The distribution of number of plasmids detected per a single *S. aureus* isolate applying TENS and PFGE methods. Each colored slice in the pie chart refers to counts of isolates with particular plasmid numbers. In case the same plasmid size were detected by both methods, only one plasmid was counted.

**FIGURE 3:**
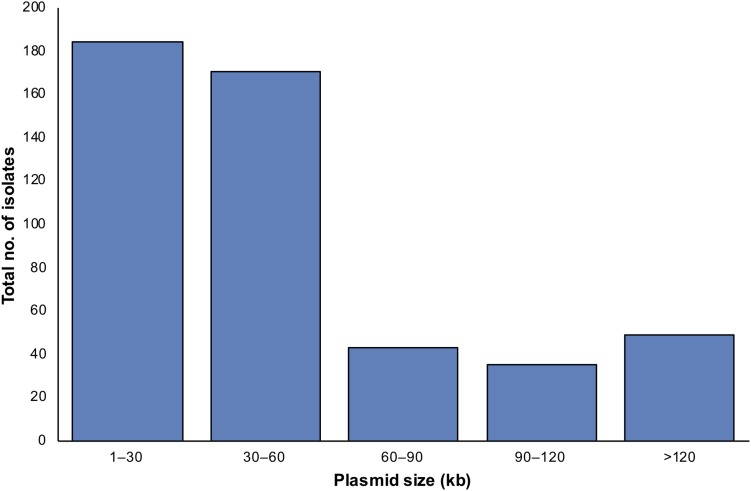
The distribution of detected plasmids in *S. aureus* isolates according to plasmid sizes applying TENS and PFGE methods. In case the same plasmid size were detected by both methods, only one plasmid was counted.

Applying both methods, all 222 *S. aureus* strains contained plasmids, with an average of four plasmids per isolate (Figure 2). Small-to-medium sized plasmids (1–60 kb) were most abundant among the isolates (Figure 3). For example, 184/222 of *S. aureus* strains contained 1–30 kb plasmids and 170/222 isolates harbored 30–60 kb plasmids. On the other hand, larger plasmids were less common among isolates; 43/222 of *S. aureus* trains harbored 60–90 kb plasmids, 35/222 contained 90–120 kb plasmids, and 49/222 harbored > 120 kb plasmids (Figure 3).

Using PFGE, the highest prevalence of large plasmids (>60 kb) consecutively was; turkey isolates (12/22), chicken liver (15/29), chicken (27/55), pork (19/42), beef liver (19/43), beef (8/24), and 36 chicken gizzard (1/7) (Table 1). Furthermore, PFGE method was able to detect plasmids of size up to 336 kb (data not shown).

### Plasmid *rep* Types

**FIGURE 4:**
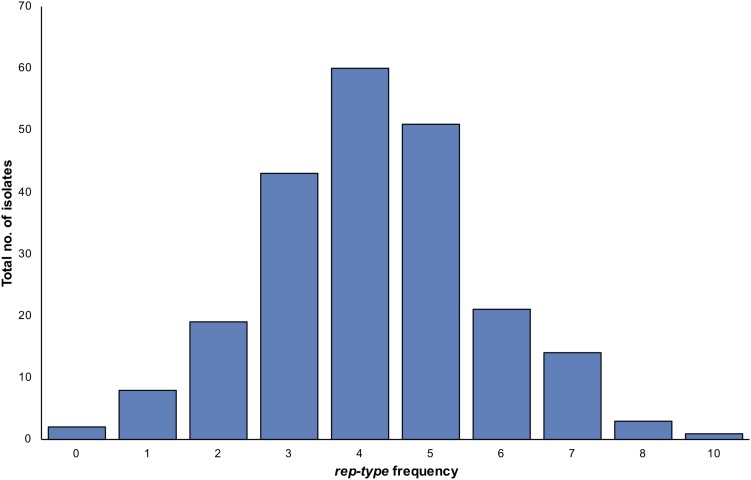
The variation in numbers of plasmid *rep* types per a single *S. aureus* isolate. The frequency refers to counts of isolates with particular *rep* type numbers. Here, more *rep* types were seen than the number of isolates as some isolates contained more than one *rep* type.

A total of 26 *rep* types (1, 2, 3, 4, 5, 6, 7, 7b, 8, 9, 10, 10b, 11, 12, 13, 14, 15, 16, 17, 18, 19, 20, 21, 22, 23, 24) were tested on the 222 *S. aureus* isolates (Supplementary Table S1). Seventeen *rep* types were detected among isolates while nine *rep* types were absent. When investigating the occurrence of a *rep* type on a single isolate, several *S. aureus* isolates harbored more than one *rep* type (Figure 4). One *S. aureus* isolates contained up to 10 *rep* types, while the majority of isolates were carrying four different *rep* types (Figure 4). Based on the assumption of one *rep* type per plasmid, some isolates showed more plasmids than *rep* types and vice versa. The most dominant *rep* types among *S. aureus* strains were *rep*_10_ and*rep_7_* (*n* = 164), *rep*_21_ (*n* = 125), *rep*_12_ (*n* = 99), *rep_7__*b*_* (*n* = 77), *rep*_13_ (*n* = 62), *rep_16_ (n* = *55), rep_22_* (*n* = 43), and *rep*_20_ (*n* = 40). Other *rep* types had a lower prevalence including *rep*_5_ (*n* = 31), *rep*_19_ (*n* = 19), *rep*_15_ (*n* = 18), *rep*_6_ (*n* = 17), *rep*_9_ (*n* = 9), *rep*_1_ (*n* = 3), *rep*_3_ (*n* = 1), and *rep_10__*b*_* (*n* = 1). Positive amplicons were not identified for *rep_2_, rep_4_*, *rep*_8_, *rep*_11_, *rep*_14_, *rep*_17_, rep*_18_*, *rep*_23_ or *rep*_24_.

### Distribution of *rep* Types According to Meat Origin

To test whether an association exists between *S. aureus rep*-types and meat origin, a chi-square test of independence was performed as described in the section “Materials and Methods.” Due to small sample size, chicken gizzard isolates were eliminated (which was *n* = 22 *rep*s of any type), as well as rep 1 (*n* = 3), rep 3 (*n* = 1), rep 9 (*n* = 9) and rep 10b (*n* = 1) isolates of any meat origin. Distribution of rep-types was found not to be independent of meat origin (*X*^2^ = 232.3, df = 60, *P* < 0.0001).

Based on the results of the chi-square test of independence we wondered whether the significance of the test was due to (1) a rather uniform departure from expected across all cells of the test (cell = rep-type by meat-type), or (2) a situation where a few cells departed greatly from expected values, but most did not. To this end we created a heat-map depicting deviation of individual chi-square cell values to use as a *visual guide* (Table 2). The chi-square cell values are those which we had summed to give the chi-square statistic [i.e., the cell value is (*o_i_*−*e_i_*)^2^/*e_i_*]. Approximately 75% of the test of independence chi-square value (i.e., *X*^2^ = 232.3) was generated by about 27% (*n* = 21) of the individual chi square cell values. Thus, deviation from expected cell values did not appear to be uniformly distributed across table cells.

**TABLE 2:** Data used for the chi-square test of independence between rep-type and meat origin.

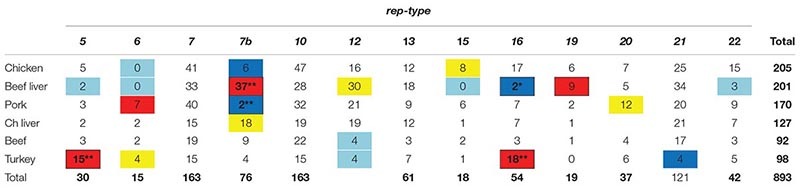

Observed occurrence of *rep*-types in meats of different origins is shown. Meat origin and rep-type were shown not to be independent (*X*^2^ = 232.3, df = 60). The color overlay is a heat map visualizing cell departure from expected; they total 75% of the *X*^2^ = 232.3 value. Red denotes that a specific chi-square element (o*_*i*_* – e*_*i*_*)^2^/e*_*i*_* > 5, and yellow > 3 where the observed value was greater than expected. Dark-blue denotes (o*_*i*_* – e*_*i*_*)^2^/e*_*i*_* > 5, and light-blue > 3 where observed value was less than expected. **Denotes significant departures from expected using a familywise error of α = 0.05 based on the Holm–Bonferroni method. This test limits the number of cells that can have very small expected values, so *rep* types that were rare (1, 3, 9, 10b), and the meat type with few samples (chicken gizzards) were eliminated.

The visual results led us to test for significant departure from expect separately in each of the 27 cells identified in the heat-map. We used a binomial model based on the expected value of a cell to calculate the mean and standard error, and the Holm–Bonferroni method to control the familywise error associated with multiple comparisons (Holm, 1979). We used a familywise error of α = 0.05). This is a conservative test (prone to Type I errors: false negative), but slightly less so that the Bonferroni test (Holm, 1979). Even so, significant departures from expected were seen for *rep* 7b in beef liver, *rep* 7b in pork, *rep* 5 in turkey, and *rep* 16 in turkey. If the familywise error is relaxed to α = 0.06 then *rep* 16 in beef lever is also significant.

**FIGURE 5:**
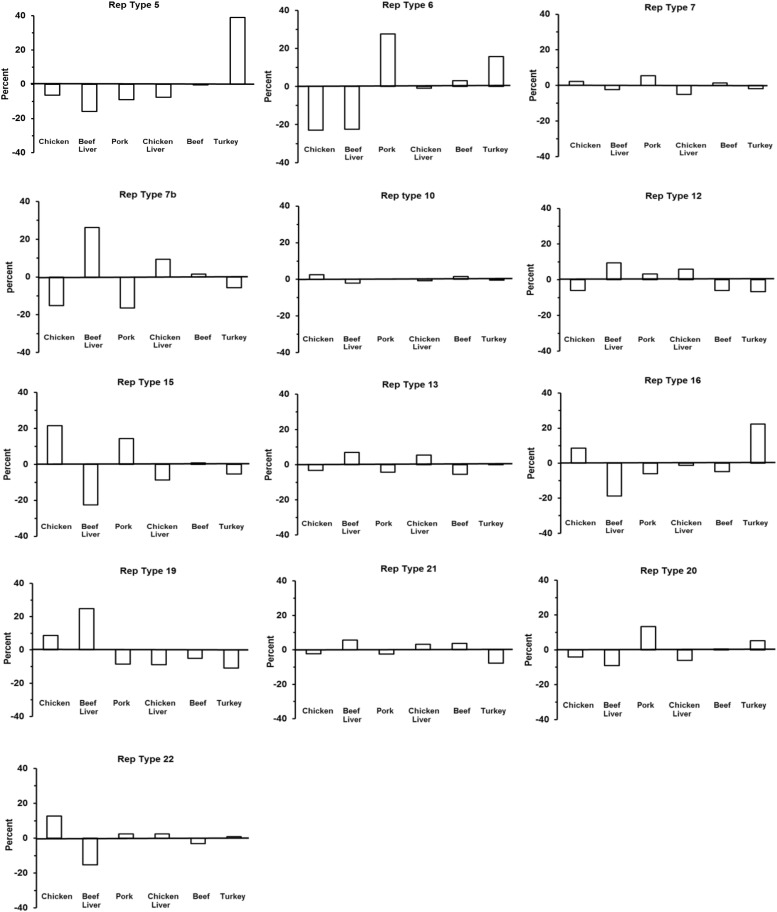
The distribution of 13 *rep* families in *S. aureus* from six different meat sources. This chart is a visual representative for the chi-square results in Table 2.

There were significant differences between some *rep* types and observed and expected frequencies for a certain meat origin, thus indicating a strong relationship (Table 2) (Figure 5). For example, a large, positive difference was observed for the following *rep* types and meat origins: *rep*_5_ and *rep*_16_, turkey*; rep_7__*b*_* and *rep*_19_, beef liver; *rep*_6_, pork; and *rep_7__*b*_*, chicken liver. Similarly, the absence of *rep* types in a specific meat origin was not random but instead more likely due to a strong interdependency between *rep* type and meat origin. Examples that support this statement include the absence of the following *rep* groups from selected meat origins: *rep_7__*b*_* in pork and chicken isolates; *rep*_16_ in beef liver; *rep*_21_ in turkey; and *rep*_22_ and *rep*_15_ in beef liver isolates. Some *rep* types exhibited a reduced relationship (or lack of relationship) with meat sources including *rep*_12_ (beef liver; turkey), *rep*_15_ (chicken), *rep*_20_ (pork), *rep*_6_ (turkey; chicken and beef liver isolates), *rep*_5_ (beef liver), and *rep_7__*b*_* (beef). Although *rep* types assigned to *rep*_7_ and *rep*_10_ occurred frequently in all meat types, there was not a significant association with a particular meat origin. Similarly, the frequency of *rep*_13_ was equal for the different meat types.

### Distribution of Antimicrobial Resistance and Virulence Genes in *rep* Types

The 222 *S. aureus* strains used in this study were previously screened for antimicrobial susceptibility to 16 different antibiotics (Abdalrahman et al., 2015a, b; Abdalrahman and Fakhr, 2015). Resistance of these 222 *S. aureus* strains to the following twelve antimicrobials (azithromycin, ciprofloxacin, gentamicin, oxacillin, tetracycline, vancomycin, trimethoprim/sulfamethazole, clindamycin, penicillin, erythromycin, rifampin, and chloramphenicol) were reassessed following the most recent CLSI published breakpoints to determine any possible association with rep types (CLSI, 2019). The distribution of antimicrobial resistance in these 222 *S. aureus* isolates was as follows: penicillin, Pen^R^ (*n* = 159), tetracycline, Tet^R^ (*n* = 143), azithromycin, Azm^R^ (*n* = 107), erythromycin, Ery^R^ (*n* = 105), oxacillin, Oxa^R^ (*n* = 88), ciprofloxacin, Cip^R^ (*n* = 68), vancomycin, Van^R^ (*n* = 66), gentamicin, Gen^R^ (*n* = 62), rifampin, Rif^R^ (*n* = 50), clindamycin, Cli^R^ (*n* = 48), trimethoprim/sulfamethoxazole, Sxt^R^ (*n* = 37), and chloramphenicol, Chl^R^ (*n* = 28) (Abdalrahman et al., 2015a, b; Abdalrahman and Fakhr, 2015). Furthermore, these isolates were evaluated for the presence of 18 genes encoding toxins including hemolysins (*hla, n* = 195; *hld, n* = 195; *hlb, n* = 95), enterotoxins (*sei*, *n* = 45; *seg, n* = 31; *seh, n* = 15; *sej, n* = 2; *sea, n* = 1; *seb, n* = 1; *sec, n* = 1; *sed, n* = 1; *see; n* = 1), toxic shock syndrome toxin-1 (*tst, n* = 9), leucocidins (*lukE-lukD, n* = 73; *lukM, n* = 0), Panton-Valentine leucocidin (PVL) (*lukS-lukF, n* = 8), and exfoliative toxins (*eta* and *etb, n* = 0).

**FIGURE 6:**
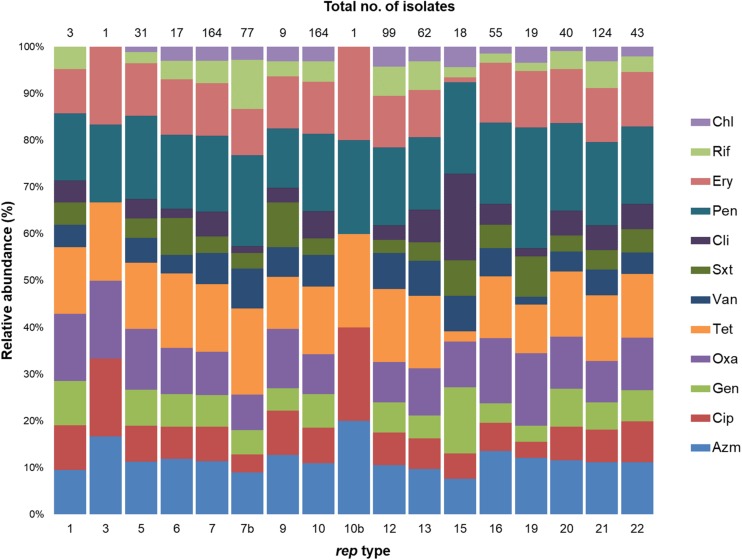
Stacked column bar graph showing the relative abundance of *S. aureus* isolates with antimicrobial resistance according to *rep* type. Antimicrobial resistance is differentiated by color as shown.

The distribution of antimicrobial resistance genes in *rep* types is shown in Figure 6. All *rep*_1_ isolates were Oxa^R^ Pen^R^ Tet^R^, but were sensitive to chloramphenicol. In isolates with *rep*_5_ amplicons, 97% were Pen^R^, and most were also resistant to Tet^R^ (77%), Oxa^R^ (71%), Ery^R^ Azm^R^ (61%). Strains with *rep*_6_ plasmids exhibited resistance to Pen^R^ Tet^R^ (94%) and Ery^R^ Azm^R^ (71%). Similarly, 75 and 67% of *rep*_7_ isolates were resistant to Pen^R^ and Tet^R^, respectively. Over 50% of isolates with *rep_7__*b*_* amplicons were Pen^R^ Tet^R^, with lower levels of resistance for other antimicrobial agents. Furthermore, *rep_7__*b*_* was frequently identified in isolates resistant to Azm, Oxa, and Pen (89%); Ery, and Tet (78%); and Cip, and Sxt (67%). The *rep*_10_ and *rep*_12_ plasmids exhibited high levels of resistance to Pen (70–72%) and Tet (66–67%). *rep*_13_ exhibited Pen^R^ Tet^R^. All *rep*_15_ strains were Pen^R^ and also showed resistance to Cli^R^ (94%), and Gen^R^ (72%). *rep*_16_ amplicons were highly resistant to Pen (84%), and also demonstrated 62-67% resistance to Oxa, Azm, Tet, and Ery. Isolates containing *rep*_19_ were Pen^R^ (79%), while resistance to other antibiotics was less frequent. Isolates with *rep*_20_ plasmids were Pen^R^ (98%), Tet^R^ (73%), and Azm^R^ Ery^R^ (60%). For isolates with *rep*_21_ plasmids, Pen^R^ (93%), were predominant, followed by resistance to Tet (79%), Ery (65%), and Oxa Ery (63%).

Although each *rep* type had a distinct resistance profile, Pen^R^ was common and Chl^R^, Rif^R^ and Van^R^ were infrequent. Among Azm^R^ isolates, the most dominant types were *rep*_9_ (89%); *rep*_6_ (71%); and *rep*_5_,*rep_16_*, *rep*_20_, and*rep_22_* (60–65%). For isolates resistant to Cip and Sxt, *rep*_9_ was predominant (67% Cip^R^ Sxt^R^), and *rep*_15_ was prevalent for Cli^R^ (97%) and Gen^R^ (72%). Oxacillin resistance was common in *rep*_9_ (89%), *rep*_5_ (71%), *rep*_16_ (67%), and *rep*_22_ (63%) amplicons. The *rep*_6_ amplicon was predominant among Tet^R^ and Oxa^R^ isolates (94% and 88%); while *rep*_15_ and *rep*_19_ were rare in Tet^R^ (11%) isolates. In Ery^R^ isolates, *rep*_9_ occurred at a high frequency (78%) compared to other *rep* types.

**FIGURE 7:**
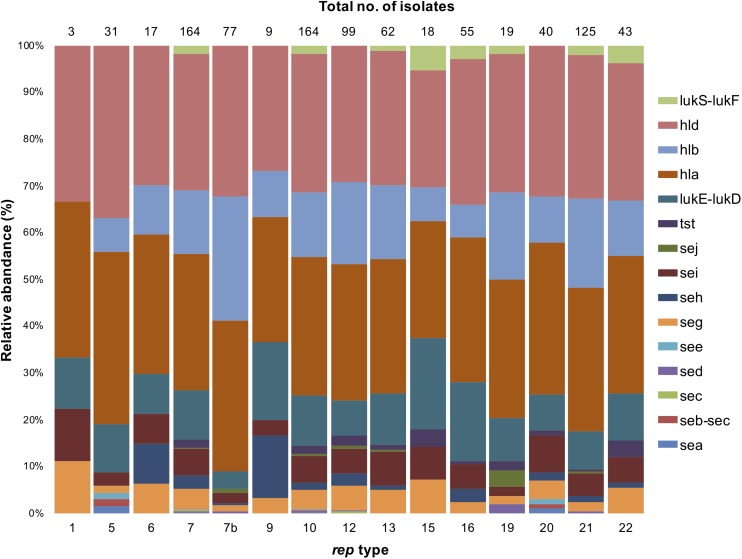
Stacked column bar graph showing the relative abundance of virulent *S. aureus* isolates according to the *rep* type. Isolates are denoted by different colors according to toxin genes as shown.

In general, toxin genes were relatively rare in *S. aureus* except for those encoding hemolysin; *hla and hld* were predominant among all *rep* types, except *rep*_3_ and *rep_10__*b.*_* (Figure 7). While the distribution of *hla* and *hld* in *rep* isolates was similar (74–100%), *hlb* was more abundant in *rep_7__*b*_* isolates (78%). Only isolates containing *rep*_5_,*rep_7_*, *rep*_10_ and *rep*_20_ were positive for enterotoxin genes *sea*, *seb*, and *see* (Figure 7). The *sec* gene was present in *rep*_7_ and *rep*_12_, *sed* was identified in *rep_7__*b*_*, *rep*_10_,*rep_12_*, *rep*_19_, and *rep*_21_, and *sej* was in isolates containing *rep*_7_, *rep_7__*b*_*, *rep*_10_, *rep_12__–__13_*, *rep*_19_, and *rep*_21_. Interestingly, *seg* and *sei* were identified in all *rep* types except *rep*_3_ and *rep_10__*b*_*, and *seh* was present in all *rep* isolates with the exception of *rep*_1_,*rep_3_*, *rep*_5_, *rep_10__*b*_*, *rep*_15_, and *rep*_19_.

The toxic shock syndrome toxin 1 gene (*tst)* was identified in *S. aureus* isolates with *rep*_7_, *rep*_10_, *rep_12__–__13_*, *rep_15__–__16_*, *rep*_19_, and *rep_20__–__22_* amplicons. The leucocidin genes, *lukE-lukD*, were associated with all *rep* types but *rep*_3_ and *rep_10__*b*_.* The *rep*_9_,*rep_15_*, and *rep*_16_ plasmids were the most prevalent types carrying *lukE-lukD* (61, 56, and 53%, respectively). The PVL genes, *lukS-lukF*, were found exclusively in *S. aureus* isolates containing *rep*_7_, *rep*_10_, *rep*_13_, *rep_15__–__16_*, *rep*_19_, and *rep_21__–__22_*. It is important to note that while these PVL and *lukS-lukF* genes may be associated with strains carrying plasmids with the above-mentioned rep-types, these toxin genes may not be carried on these plasmids.

## Discussion

Our understanding of *S. aureus* plasmids is biased toward clinical strains, and limited information is available regarding plasmids harbored by foodborne *S. aureus*, especially in retail meats. Furthermore, no standardized typing approach is available to classify plasmids from various bacterial species. In this study, a PCR-based approach targeting *rep* genes was used to investigate plasmids in 222 *S. aureus* isolated from seven different retail meats; the aim was to expand the current plasmid classification system for gram-positive bacteria and to analyze the distribution and prevalence of these plasmids.

Many genes associated with *S. aureus* survival and persistence are plasmid-encoded, including those associated with antimicrobial resistance, biofilm formation and toxin production (Bukowski et al., 2019). In this study, the plasmids in *S. aureus* strains from retail meats exhibited diverse *rep* types, which might confer a selective advantage. In other words, the presence of multiple plasmids encoding different virulence factors or resistance traits provide a selective advantage for the bacterial host in different environments (Carattoli, 2013). Some isolates contained plasmids that were not assigned to a *rep* type; this might be due to the presence of novel or mutated *rep* genes that were not be identified by the primers (Yano et al., 2016). On the other hand, some strains harbored more *rep* sequences than number of plasmids; this can be explained by plasmid integration or fusion with another plasmid (Monk and Foster, 2012), existence of more than one plasmid of a given size, or failure to identify a plasmid by alkaline lysis and PFGE. While the occurrence of *rep* genes likely indicates the presence of a particular plasmid type, definite conclusions cannot be derived and caution must be applied. Future studies using a whole genome sequencing approach are therefore recommended.

The majority of isolates in the present study harbored plasmids > 20 kb; large plasmids are key contributors to HGT and host adaptation to different environments (Haaber et al., 2017). Indeed, our study revealed a high occurrence of large plasmids in *S. aureus* populations isolated from retail meats. Large plasmids often encode a diverse range of virulence genes and genetic modules to ensure their stability within the host and facilitate transfer to other bacteria (Shearer et al., 2011). Recent studies demonstrated that larger plasmids undergo mutations in genes encoding plasmid replication initiation proteins to increase adaptation to bacterial hosts (Yano et al., 2016) or may acquire stabilizing traits from other resident plasmids (Loftie-Eaton et al., 2016). It is important to mention that *S. aureus* carries an efficient restriction-modification system to prevent the entry of unrecognizable DNA (Waldron and Lindsay, 2006); however, conjugative plasmids may avoid the *S. aureus* restriction system by losing restriction sites (Roberts et al., 2013). Although they play an important role in host survival, virulence, and gene exchange, the larger plasmids in *S. aureus* remain poorly studied. For example, a recent study showed that only the origin of replication in small plasmids was required for transfer to co-resident conjugative plasmids (O’Brien et al., 2012).

Our study shows that *rep* families are diverse among *S. aureus* meat isolates, and some *rep* types were more abundant than others. The two dominant *rep* families, *rep*_7_ and *rep*_10_, had no significant association with a particular meat product. These findings agree with a European study that reported identical *rep* families in *S. aureus* strains from humans, animals, and food (Lozano et al., 2012). Many commensal *S. aureus* strains can be shared between humans and food animals that can serve as reservoirs for *rep* plasmid families (Smith, 2015). Interestingly, the *rep_7__*b*_* and *rep*_12_ types were fairly abundant in this study and were significantly linked to *S. aureus* isolated from liver. In the slaughterhouse, edible offal (internal organs) are prepared in the early stage of slaughtering, and many studies have suggested improper human processing and handling as primary sources for offal contamination (Im et al., 2016; Rouger et al., 2017). It is important to note that typing of *S. aureus* isolates was not conducted in this current study, and this is critical to accurately track the source of identified plasmids in retail meats.

In this study, selected *rep* families showed a remarkable specificity for certain meat origins, thus indicating that food animals can serve as reservoirs for these plasmids. We found that *rep*_5_ and *rep*_16_ were dominant types in turkey, *rep*_6_ in pork and turkey, *rep*_15_ in chicken, and *rep*_20_ in pork isolates. *S. aureus* strains occur as commensal organisms on the skin, nose and mucous membranes of food animals (Lozano et al., 2012) and food production animals are often colonized with specific *S. aureus* strains (Wall et al., 2016). When exposed to the selective pressure of antimicrobial use, *S. aureus* adapts by acquiring genes from other organisms and can become more persistent in the livestock production system. This was supported by the observed correlation between the above-mentioned *rep* families and isolates resistant to antimicrobials commonly used in food animals (e.g., Sxt and Tet). Another interesting result in the current study was the link between the *rep*_6_ type and multiple food animals, whereas other *rep* families were exclusive to a single source. In contrast to other *rep* types, the *rep*_6_ family is prevalent in broad-host-range plasmids (Jensen et al., 2010), which confirms the role of plasmids in distributing genes in *S. aureus* isolates. Although our study was restricted to *S. aureus* strains from retail meats, it suggests the potential role of food animals as a source for *S. aureus* plasmids in the meat pyramid.

Meat products may also become contaminated with *S. aureus* strains that harbor plasmids that originated from other gram-positive organisms. Surprisingly, *rep*_3_ and *rep*_12_ plasmid families were detected in the *S. aureus* strains analyzed in this study. These *rep* families were previously found in *Bacillus* spp. and are thought to have a narrow host range (Jensen et al., 2010). It is also noteworthy that the rep*_1_* and*rep_9_* amplicons, which were previously detected in few *S. aureus* strains, are naturally occurring in *Enterococcus* spp. (Jensen et al., 2010; Lozano et al., 2012). Plasmid transfer from *Enterococcus* or *Bacillus* spp. to *S. aureus* is not unusual and has been observed in previous studies (Gryczan et al., 1978; Zhu et al., 2010). Moreover, *S. aureus* produces a peptide known to induce bacterial clumping, which initiates HGT of *Enterococcus* spp. plasmids (Clewell et al., 1985). Other factors can also promote HGT between *S. aureus* strains such as the occurrence of other bacterial species with *S. aureus* strains. Both *Bacillus* and *Enterococcus* spp. are widespread in the farm environment and can be disseminated to the slaughterhouse by incoming animal carcasses or meat industry workers (Gutiérrez et al., 2012); HGT can occur in biofilms that form within meat production facilities or during colonization of livestock dermis or nasal cavities (Coimbra-E-Souza et al., 2019). Furthermore, the aforementioned plasmid families could harbor biological features that favor HGT into *S. aureus* strains. For example, the *rep*_12_ amplicon type is found in *B. thuringiensis* pBMB67 plasmids that encode conjugal transfer genes (Chao et al., 2007; Shintani et al., 2015), while the *rep*_9_ type was detected in *E. faecalis* conjugative plasmids (Jensen et al., 2010). Our findings support the contention that intergeneric transfer of plasmids occurs in the meat production chain under selection pressure. Thus, further research is needed to understand the conditions that promote plasmid transfer – if any – in the meat production industry with the aim of limiting the dissemination of antimicrobial resistance and virulence genes in meat-associated bacteria.

An association between *rep* families and antimicrobial resistance was observed in our study. McCarthy and Lindsay (2012) found a strong association between plasmid groups containing the *rep*_15_ sequence and the occurrence of *tetK*; whereas another study found the same *rep* type in Tet^R^ *S. aureus* isolates (Lozano et al., 2012). In our study, there was a high prevalence of the *rep*_15_ type in Tet^R^ strains. Furthermore, the association between *rep*_6_ and*rep_15_* types and Sxt^R^, the *rep_7__*b*_* type and Cip^R^, and the *rep*_19_ type and β-lactam resistance was in agreement with other studies and may indicate that the antimicrobial genes are plasmid-encoded (Lozano et al., 2012; McCarthy and Lindsay, 2012). On the other hand, many *rep* families were associated with rifamycin-resistant *S. aureus*. Although Rif resistance generally occurs due to chromosomal mutations (Zhou et al., 2012), it can also be plasmid-mediated (Arlet et al., 2001; Girlich et al., 2001). The mechanistic basis of Rif^R^ in our isolates and the role of *rep* types are worthy of future study.

The excessive use of antibiotics in humans and food animals is a driving force for spreading and maintaining virulent strains and their plasmids (Zurfluh et al., 2014; Wall et al., 2016). Thereby, knowledge of plasmid types carried by resistant strains is pivotal in controlling or impacting plasmid-mediated dissemination of antibiotic resistance. Nevertheless, our data must be interpreted with caution because the resistance profiles for *S. aureus* isolates were phenotypic; further analysis is required to confirm the association between certain *rep* families and the resistance of *S. aureus* in meat products.

The application of the *rep* typing scheme is a valuable tool for improving knowledge of plasmid dissemination and distribution in *S. aureus* inhabiting various environments, particularly the meat production system. Furthermore, links between *rep* families and specific genes would be helpful in future efforts designed to limit the dissemination of virulent strains in the meat pyramid. This study has confirmed the presence of plasmids with diverse sizes in multidrug-resistant *S. aureus*, which indicates that meat products might play a possible role in disseminating these strains and their plasmids to human consumers. Moreover, our results suggest that multiple sources and factors contribute to the spread and maintenance of plasmid-bearing *S. aureus* strains in the food chain.

## Data Availability Statement

All datasets generated for this study are included in the article/Supplementary Material.

## Author Contributions

MF worked on the research design. LN and NR were responsible for the experimental procedures. HW and LN did the statistical analysis. LN and MF prepared the manuscript.

## Conflict of Interest

The authors declare that the research was conducted in the absence of any commercial or financial relationships that could be construed as a potential conflict of interest.

## Supplementary Material

FIGURE S1 Detection of plasmids in *S. aureus* isolates by Pulsed Field Gel Electrophoresis (PFGE). Red arrows in lanes 2, 5, 9, and 10 show large plasmids approximately 152 kb (lanes 2, 5) and 120 kb (lanes 9, 10). Yellow arrows indicate plasmids approximately 70 kb (lane 7) and 65 kb (lane 11). Small plasmids approximately 20 kb are shown in lanes 2, 3, 5, 9, and 10 (blue arrows). Lane 9 contains the *Salmonella* serovar Braenderup H9812 marker.

TABLE S1 *rep* primers and multiplexes used for PCR.

## References

[B1] Aarestrup F. M. Wegener H. C. Collignon P. (2008). Resistance in bacteria of the food chain: epidemiology and control strategies. *Expert Rev. Anti. Infect. Ther.* 6 733–750. 10.1586/14787210.6.5.733 18847409

[B2] Abdalrahman L. S. Fakhr M. K. (2015). Incidence, antimicrobial susceptibility, and toxin genes possession screening of *Staphylococcus aureus* in retail chicken livers and gizzards. *Foods* 4 115–129. 10.3390/foods4020115 28231192 PMC5302321

[B3] Abdalrahman L. S. Stanley A. Wells H. Fakhr M. K. (2015a). Isolation, virulence, and antimicrobial resistance of methicillin-resistant *Staphylococcus aureus* (MRSA) and methicillin sensitive *Staphylococcus aureus* (MSSA) Strains from Oklahoma Retail Poultry Meats. *Int. J. Environ. Res. Public Health* 12 6148–6161. 10.3390/ijerph120606148 26035662 PMC4483693

[B4] Abdalrahman L. S. Wells H. Fakhr M. K. (2015b). *Staphylococcus aureus* is more prevalent in retail beef livers than in pork and other beef cuts. *Pathogens* 4 182–198. 10.3390/pathogens4020182 25927961 PMC4493469

[B5] Argudín M. A. Mendoza M. C. Rodicio M. R. (2010). Food poisoning and *Staphylococcus aureus* enterotoxins. *Toxins* 2 1751–1773. 10.3390/toxins2071751 22069659 PMC3153270

[B6] Arlet G. Nadjar D. Herrmann J. L. Donay J. L. Lagrange P. H. Philippon A. (2001). Plasmid-mediated rifampin resistance encoded by an arr-2-like gene cassette in *Klebsiella pneumoniae* producing an ACC-1 class C beta-lactamase. *Antimicrob. Agents Chemother.* 45 2971–2972. 10.1128/aac.45.10.2971-2972.2001 11583008 PMC90768

[B7] Baines S. L. Jensen S. O. Firth N. da Silva A. Seemann T. Carter G. P. (2019). Remodeling of pSK1 family plasmids and enhanced chlorhexidine tolerance in a dominant hospital lineage of methicillin-resistant *Staphylococcus aureus*. *Antimicrob. Agents Chemother.* 63:e002356-18. 10.1128/AAC.02356-18 30783008 PMC6496109

[B8] Bukowski M. Piwowarczyk R. Madry A. Zagorski-Przybylo R. Hydzik M. Wladyka B. (2019). Prevalence of antibiotic and heavy metal resistance determinants and virulence-related genetic elements in plasmids of *Staphylococcus aureus*. *Front. Microbiol.* 10:805. 10.3389/fmicb.2019.00805 31068910 PMC6491766

[B9] Caddick J. M. Hilton A. C. Rollason J. Lambert P. A. Worthington T. Elliott T. S. J. (2005). Molecular analysis of methicillin-resistant *Staphylococcus aureus* reveals an absence of plasmid DNA in multidrug-resistant isolates. *FEMS Immunol. Med. Microbiol.* 44 297–302. 15907452 10.1016/j.femsim.2004.12.014

[B10] Carattoli A. (2013). Plasmids and the spread of resistance. *Int. J. Med. Microbiol.* 303 298–304. 10.1016/j.ijmm.2013.02.001 23499304

[B11] Chao L. Qiyu B. Fuping S. Ming S. Dafang H. Guiming L. (2007). Complete nucleotide sequence of pBMB67, a 67-kb plasmid from *Bacillus thuringiensis* strain YBT-1520. *Plasmid* 57 44–54. 10.1016/j.plasmid.2006.06.002 16901541

[B12] Clewell D. B. An F. Y. White B. A. Gawron-Burke C. (1985). *Streptococcus faecalis* sex pheromone (cAM373) also produced by *Staphylococcus aureus* and identification of a conjugative transposon (Tn918). *J. Bacteriol.* 162 1212–1220. 10.1128/jb.162.3.1212-1220.1985 2987186 PMC215906

[B13] CLSI, (2019). *Performance Standards for Antimicrobial Susceptibility Testing*, 29th Edn, Wayne, PA: Clinical and Laboratory Standards Institute.

[B14] Coimbra-E-Souza V. Rossi C. C. Jesus-de Freitas L. J. Brito M. A. V. P. Laport M. S. Giambiagi-deMarval M. (2019). Short communication: diversity of species and transmission of antimicrobial resistance among *Staphylococcus* spp. isolated from goat milk. *J. Dairy Sci.* 102 5518–5524. 10.3168/jds.2018-15723 30928272

[B15] DeLeo F. R. Chambers H. F. (2009). Reemergence of antibiotic-resistant *Staphylococcus aureus* in the genomics era. *J. Clin. Invest.* 119 2464–2474. 10.1172/JCI38226 19729844 PMC2735934

[B16] Fritz S. A. Epplin E. K. Garbutt J. Storch G. A. (2009). Skin infection in children colonized with community-associated methicillin-resistant *Staphylococcus aureus*. *J. Infect.* 59 394–401. 10.1016/j.jinf.2009.09.001 19747505 PMC2788074

[B17] Girlich D. Poirel L. Leelaporn A. Karim A. Tribuddharat C. Fennewald M. (2001). Molecular epidemiology of the integron-located VEB-1 extended-spectrum beta-lactamase in nosocomial enterobacterial isolates in Bangkok, Thailand. *J. Clin. Microbiol.* 39 175–182. 10.1128/jcm.39.1.175-182.2001 11136767 PMC87698

[B18] Gryczan T. J. Contente S. Dubnau D. (1978). Characterization of *Staphylococcus aureus* plasmids introduced by transformation into *Bacillus subtilis*. *J. Bacteriol.* 134 318–329. 10.1128/jb.134.1.318-329.1978 418061 PMC222249

[B19] Gutiérrez D. Delgado S. Vázquez-Sánchez D. Martínez B. Cabo M. L. Rodríguez A. (2012). Incidence of *Staphylococcus aureus* and analysis of associated bacterial communities on food industry surfaces. *Appl. Environ. Microbiol.* 78 8547–8554. 10.1128/AEM.02045-12 23023749 PMC3502933

[B20] Haaber J. Penadés J. R. Ingmer H. (2017). Transfer of antibiotic resistance in *Staphylococcus aureus*. *Trends Microbiol.* 25 893–905. 10.1016/j.tim.2017.05.011 28641931

[B21] Holm S. (1979). A simple and sequential rejective multiple test procedure. *Scand. J. Statist.* 6 65–70.

[B22] Im M. C. Seo K. W. Bae D. H. Lee Y. J. (2016). Bacterial quality and prevalence of foodborne pathogens in edible offal from slaughterhouses in Korea. *J. Food Prot.* 79 163–168. 10.4315/0362-028X.JFP-15-251 26735045

[B23] Jackson C. R. Davis J. A. Barrett J. B. (2013). Prevalence and characterization of methicillin-resistant *Staphylococcus aureus* isolates from retail meat and humans in Georgia. *J. Clin. Microbiol.* 51 1199–1207. 10.1128/JCM.03166-12 23363837 PMC3666775

[B24] Jensen L. B. Garcia-Migura L. Valenzuela A. J. S. Løhr M. Hasman H. Aarestrup F. M. (2010). A classification system for plasmids from enterococci and other Gram-positive bacteria. *J. Microbiol. Methods* 80 25–43. 10.1016/j.mimet.2009.10.012 19879906

[B25] Jensen S. O. Lyon B. R. (2009). Genetics of antimicrobial resistance in *Staphylococcus aureus*. *Future Microbiol.* 4 565–582. 10.2217/fmb.09.30 19492967

[B26] Kadariya J. Smith T. C. Thapaliya D. (2014). *Staphylococcus aureus* and staphylococcal food-borne disease: an ongoing challenge in public health. *Biomed. Res. Int.* 2014:827965. 10.1155/2014/827965 24804250 PMC3988705

[B27] Kadlec K. Schwarz S. (2010). Identification of a plasmid-borne resistance gene cluster comprising the resistance genes erm(T), dfrK, and tet(L) in a porcine methicillin-resistant *Staphylococcus aureus* ST398 strain. *Antimicrob. Agents Chemother.* 54 915–918. 10.1128/AAC.01091-09 20008780 PMC2812154

[B28] Kluytmans J. van Leeuwen W. Goessens W. Hollis R. Messer S. Herwaldt L. (1995). Food-initiated outbreak of methicillin-resistant *Staphylococcus aureus* analyzed by pheno- and genotyping. *J. Clin. Microbiol.* 33 1121–1128. 10.1128/jcm.33.5.1121-1128.1995 7615715 PMC228116

[B29] Loftie-Eaton W. Yano H. Burleigh S. Simmons R. S. Hughes J. M. Rogers L. M. (2016). Evolutionary paths that expand plasmid host-range: implications for spread of antibiotic resistance. *Mol. Biol. Evol.* 33 885–897. 10.1093/molbev/msv339 26668183 PMC4840908

[B30] Lozano C. García-Migura L. Aspiroz C. Zarazaga M. Torres C. Aarestrup F. M. (2012). Expansion of a plasmid classification system for Gram-positive bacteria and determination of the diversity of plasmids in *Staphylococcus aureus* strains of human, animal, and food origins. *Appl. Environ. Microbiol.* 78 5948–5955. 10.1128/AEM.00870-12 22685157 PMC3406130

[B31] Malachowa N. DeLeo F. R. (2010). Mobile genetic elements of *Staphylococcus aureus*. *Cell. Mol. Life Sci.* 67 3057–3071. 10.1007/s00018-010-0389-4 20668911 PMC2929429

[B32] Marasini D. Fakhr M. K. (2014). Exploring PFGE for detecting large plasmids in *Campylobacter jejuni* and *Campylobacter coli* isolated from various retail meats. *Pathogens* 3 833–844. 10.3390/pathogens3040833 25436507 PMC4282888

[B33] Marmur J. (1961). A procedure for the isolation of deoxyribonucleic acid from micro-organisms. *J. Mol. Biol.* 3 208–218.

[B34] McCarthy A. J. Lindsay J. A. (2012). The distribution of plasmids that carry virulence and resistance genes in *Staphylococcus aureus* is lineage associated. *BMC Microbiol.* 12:104. 10.1186/1471-2180-12-104 22691167 PMC3406946

[B35] McDougal L. K. Steward C. D. Killgore G. E. Chaitram J. M. McAllister S. K. Tenover F. C. (2003). Pulsed-field gel electrophoresis typing of oxacillin-resistant *Staphylococcus aureus* isolates from the United States: establishing a national database. *J. Clin. Microbiol.* 41 5113–5120. 10.1128/jcm.41.11.5113-5120.2003 14605147 PMC262524

[B36] Monk I. R. Foster T. J. (2012). Genetic manipulation of *Staphylococci*-breaking through the barrier. *Front. Cell Infect. Microbiol.* 2:49. 10.3389/fcimb.2012.00049 22919640 PMC3417578

[B37] Noormohamed A. Fakhr M. K. (2012). Incidence and antimicrobial resistance profiling of *Campylobacter* in retail chicken livers and gizzards. *Foodborne Pathog. Dis.* 9 617–624. 10.1089/fpd.2011.1074 22545960

[B38] Novick R. P. (1987). Plasmid incompatibility. *Microbiol. Rev.* 51 381–395. 3325793 10.1128/mr.51.4.381-395.1987 PMC373122

[B39] O’Brien A. M. Hanson B. M. Farina S. A. Wu J. Y. Simmering J. E. Wardyn S. E. (2012). MRSA in conventional and alternative retail pork products. *PLoS One* 7:30092. 10.1371/journal.pone.0030092 22276147 PMC3261874

[B40] Ogston A. (1882). Micrococcus poisoning. *J. Anat. Physiol.* 16 526–567. PMC1310038 17231444

[B41] Orlek A. Phan H. Sheppard A. E. Doumith M. Ellington M. Peto T. (2017). Ordering the mob: insights into replicon and MOB typing schemes from analysis of a curated dataset of publicly available plasmids. *Plasmid* 91 42–52. 10.1016/j.plasmid.2017.03.002 28286183 PMC5466382

[B42] Roberts G. A. Houston P. J. White J. H. Chen K. Stephanou A. S. Cooper L. P. (2013). Impact of target site distribution for Type I restriction enzymes on the evolution of methicillin-resistant *Staphylococcus aureus* (MRSA) populations. *Nucleic Acids Res.* 41 7472–7484. 10.1093/nar/gkt535 23771140 PMC3753647

[B43] Rouger A. Tresse O. Zagorec M. (2017). Bacterial contaminants of poultry meat: sources, species, and dynamics. *Microorganisms* 5:50. 10.3390/microorganisms5030050 28841156 PMC5620641

[B44] Sambrook J. Russell D. W. (2001). *Molecular Cloning: A Laboratory Manual*, 3rd Edn, Cold Spring Harbor, NY: Cold Spring Harbor Laboratory Press.

[B45] Scallan E. Hoekstra R. M. Angulo F. J. Tauxe R. V. Widdowson M.-A. Roy S. L. (2011). Foodborne illness acquired in the United States–major pathogens. *Emerg. Infect. Dis.* 17 7–15. 10.3201/eid1701.P11101 21192848 PMC3375761

[B46] Shahkarami F. Rashki A. Rashki Ghalehnoo Z. (2014). Microbial susceptibility and plasmid profiles of methicillin-resistant *Staphylococcus aureus* and methicillin-susceptible S. aureus. *Jundishapur J. Microbiol.* 7:e16984. 10.5812/jjm.16984 25368805 PMC4216585

[B47] Shearer J. E. S. Wireman J. Hostetler J. Forberger H. Borman J. Gill J. (2011). Major families of multiresistant plasmids from geographically and epidemiologically diverse staphylococci. *G3* 1 581–591. 10.1534/g3.111.000760 22384369 PMC3276174

[B48] Shintani M. Sanchez Z. K. Kimbara K. (2015). Genomics of microbial plasmids: classification and identification based on replication and transfer systems and host taxonomy. *Front. Microbiol.* 6:242. 10.3389/fmicb.2015.00242 25873913 PMC4379921

[B49] Smith T. C. (2015). Livestock-associated *Staphylococcus aureus*: the United States experience. *PLoS Pathog.* 11:e1004564. 10.1371/journal.ppat.1004564 25654425 PMC4412291

[B50] Sokal R. R. Rohlf R. J. (1995). *Biometry: The Principles and Practice of Statistics in Biological Research*, 3rd Edn, New York, NY: W. H. Freeman.

[B51] Waldron D. E. Lindsay J. A. (2006). Sau1: a novel lineage-specific type I restriction-modification system that blocks horizontal gene transfer into *Staphylococcus aureus* and between *S. aureus* isolates of different lineages. *J. Bacteriol.* 188 5578–5585. 10.1128/jb.00418-06 16855248 PMC1540015

[B52] Wall B. A. Mateus A. Marshall L. Pfeiffer D. U. Lubroth J. Ormel H. J. (2016). *Drivers, Dynamics and Epidemiology of Antimicrobial Resistance in Animal Production.* Rome: Food and Agriculture Organization of the United Nations.

[B53] Waters A. E. Contente-Cuomo T. Buchhagen J. Liu C. M. Watson L. Pearce K. (2011). Multidrug-resistant *Staphylococcus aureus* in US Meat and poultry. *Clin. Infect. Dis.* 52 1227–1230. 10.1093/cid/cir181 21498385 PMC3079400

[B54] Yano H. Wegrzyn K. Loftie-Eaton W. Johnson J. Deckert G. E. Rogers L. M. (2016). Evolved plasmid-host interactions reduce plasmid interference cost. *Mol. Microbiol.* 101 743–756. 10.1111/mmi.13407 27121483 PMC5024541

[B55] Zhou W. Shan W. Ma X. Chang W. Zhou X. Lu H. (2012). Molecular characterization of rifampicin-resistant *Staphylococcus aureus* isolates in a Chinese teaching hospital from Anhui, China. *BMC Microbiol.* 12:240. 10.1186/1471-2180-12-240 23082766 PMC3485161

[B56] Zhu W. Murray P. R. Huskins W. C. Jernigan J. A. McDonald L. C. Clark N. C. (2010). Dissemination of an *Enterococcus* Inc18-Like vanA plasmid associated with vancomycin-resistant *Staphylococcus aureus*. *Antimicrob. Agents Chemother.* 54 4314–4320. 10.1128/AAC.00185-10 20660665 PMC2944587

[B57] Zurfluh K. Jakobi G. Stephan R. Hächler H. Nüesch-Inderbinen M. (2014). Replicon typing of plasmids carrying bla CTX-M-1 in *Enterobacteriaceae* of animal, environmental and human origin. *Front. Microbiol.* 5:555. 10.3389/fmicb.2014.00555 25400623 PMC4214192

