# Supplementary figures and images for: Molecular Characterization of Staphylococcus aureus Plasmids Associated With Strains Isolated From Various Retail Meats
Source: Front Microbiol. 2020 Feb 19;11:223. doi: 10.3389/fmicb.2020.00223 (PMC7042431; doi:10.3389/fmicb.2020.00223)

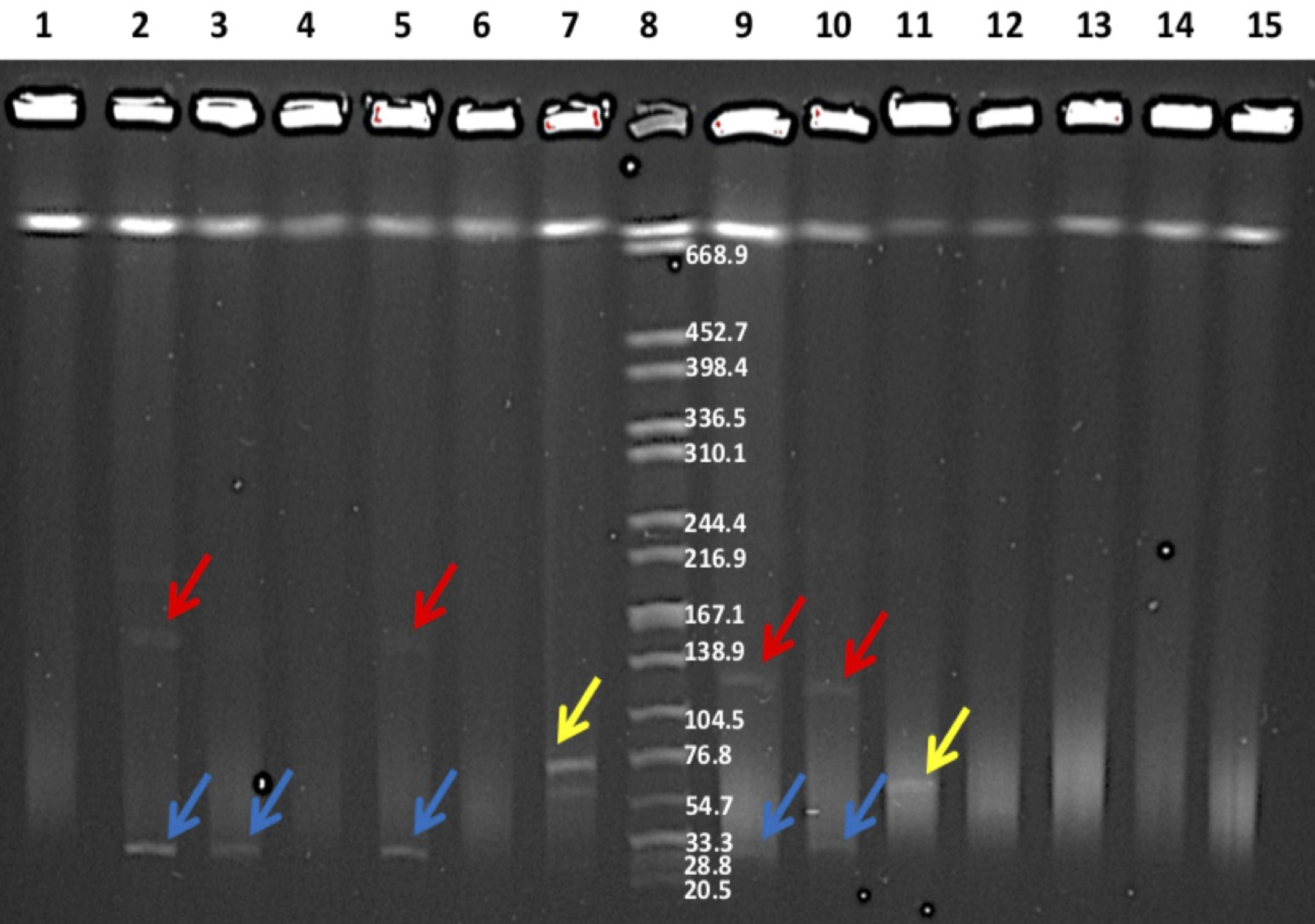

Supplement: FIGURE S1 — Detection of plasmids in S. aureus isolates by Pulsed Field Gel Electrophoresis (PFGE). Red arrows in lanes 2, 5, 9, and 10 show large plasmids approximately 152 kb (lanes 2, 5) and 120 kb (lanes 9, 10). Yellow arrows indicate plasmids approximately 70 kb (lane 7) and 65 kb (lane 11). Small plasmids approximately 20 kb are shown in lanes 2, 3, 5, 9, and 10 (blue arrows). Lane 9 contains the Salmonella serovar Braenderup H9812 marker. [file Image_1.TIFF]
