# Supplementary material for: Molecular Characterization of Staphylococcus aureus Plasmids Associated With Strains Isolated From Various Retail Meats
Source: Front Microbiol. 2020 Feb 19;11:223. doi: 10.3389/fmicb.2020.00223 (PMC7042431; doi:10.3389/fmicb.2020.00223)
Supplement: TABLE S1 — rep primers and multiplexes used for PCR. [file Table_1.docx]

Supplementary Table S1. *rep* primers and multiplexes used for PCR.

| ***rep* type** | **Multiplex PCR** | **Size (bp)** | **Multiplex Temp (°C)** | **Primers (5’🡪 3’)** | **Reference** |
| --- | --- | --- | --- | --- | --- |
|  |  |  |  |  |  |
| 1 | 1 | 624 | 56 | TCGCTCAATCACTACCAAGC  CTTGAACGAGTAAAGCCCTT | (Jensen *et al*., 2010) |
| 2 | 4 | 630 | 56 | GAGAACCATCAAGGCGAAAT  ACCAGAATAAGCACTACGTACAATCT | (Jensen *et al*., 2010) |
| 3 | 3 | 403 | 52 | CCTAATGTATATAATTTTGGTACATAT  ACATTTTCCTCAAAGAACAT | (Jensen *et al*., 2010) |
| 4 | 2 | 430 | 52 | ACTATGTCGTTGAGTCTAATGACT  AGCAAGATAGAATATTTACTTTTAAGTTT | (Jensen *et al*., 2010) |
| 5 | 6 | 257 | 52 | CTTAAATCTACMTATTCWAAAMAYATGTT  TCARCGTCAAAWGTRAACTCT | (Lozano *et al*., 2012) |
| 6 | 7 | 551 | 56 | ACGAATGAAAGATAAAGGAGTAG  TAAATTCTAGTTTGGCAATCTTAT | (Jensen *et al*., 2010) |
| 7 | 2 | 227 | 52 | AGACGTAATATGCGTRTTGA  CCAAAATAYTTYGTTTCTGG | (Jensen *et al*., 2010) |
| 7b | 7 | 729 | 56 | CTAATAGCCGGTTAGACGCAC  GACGRGARTTTCTATGTAATTCTCC | (Lozano *et al*., 2012) |
| 8 | 4 | 394 | 56 | TAGATACGACAAAAGAAGAATTACA  CCAATCATGTAATGTTACAACC | (Jensen *et al*., 2010) |
| 9 | 1 | 201 | 56 | GCTCGATCARTTTTCAGAAG  CGCAAACATTTGTCWATTTCTT | (Jensen *et al*., 2010) |
| 10 | 1 | 382 | 56 | TATAAAGGCTCTCAGAGGCT  CCAAATTCGAGTAAGAGGTA | (Jensen *et al*., 2010) |
| 10b | 3 | 200 | 52 | TAAATAAAGACTCAGGAGAAGTA  TAGCAAGTTCTCGAACTGTT | (Jensen *et al*., 2010) |
| 11 | 6 | 500 | 52 | TCTAGAATGCGTAAAAAGG  CCTTTGAAGATWGCRGTWAG | (Jensen *et al*., 2010) |
| 12 | 3 | 470 | 52 | GAGCCTATAACAGAGTACACA  CAAATATAGGCTTTGTAGTTC | (Jensen *et al*., 2010) |
| 13 | 5 | 402 | 52 | ATGATGCAATATATTAAGCA  TACCAGAATAYTTAGCCATTTC | (Jensen *et al*., 2010) |
| 14 | 2 | 164 | 52 | GAAAGYTTRGATAGYTTTGC  RTTTTGRCTTTCTTSYTTCA | (Jensen *et al*., 2010) |
| 15 | 5 | 327 | 52 | CAGTAGAAGAAAATTATAAAGAAC  GTTATGGCTGGTTTTAATAAA | (Jensen *et al*., 2010) |
| 16 | 5 | 592 | 52 | CAGGAAAACACTTCGTTTAT  CTTCTATATCACTATCATTGTCATT | (Jensen *et al*., 2010) |
| 17 | 2 | 604 | 52 | TACTAACTGTTGGTAATTCGTTAAAT  ATCAAGGACTCAACCGTAATT | (Jensen *et al*., 2010) |
| 18 | 4 | 462 | 56 | ACACCAGTCGAAATGAATTT  AGGAATATCAAGTAATTCATGAAAGT | (Jensen *et al*., 2010) |

Supplementary Table 1. (continued).

| 19 | 3 | 543 | 52 | GWGATCGCTTARAYTTATCTAT  YMTTGTTSTGGMAATTCTT | (Jensen *et al*., 2010) |
| --- | --- | --- | --- | --- | --- |
| 20 | 6 | 329 | 52 | CTGTGAATYTGGAAGTTCC  CTAAATATTTRTTYGAAGATGC | (Lozano *et al*., 2012) |
| 21 | 6 | 414 | 52 | TAGTTATCAAGCTCARARAG  GMTTSWATRTCTTTATCGCC | (Lozano *et al*., 2012) |
| 22 | 7 | 486 | 56 | TCCGTTCCCAATTCCACATTGCAA  TGCGTGCAACGGAAGTGACA | (Lozano *et al*., 2012) |
| 23 | 7 | 293 | 56 | TGGTCGTGACGCACATTACACA  CGCTATTTTCCTCCGTATCCGG | (Lozano *et al*., 2012) |
| 24 | 4 | 229 | 56 | TCACAGAACTTCAAGAATTCCC  CCTTGCATAGTYTCAACACTTTC | (Lozano *et al*., 2012) |
